# Multisystem Inflammatory Syndrome in Children with tailored therapy and six-month outcome

**DOI:** 10.1038/s41390-025-04706-6

**Published:** 2026-01-10

**Authors:** Osman Oguz Demir, Kubra Aykac, Arthur Hoi Hin Cheng, Selman Kesici, H. Hakan Aykan, Yelda Bilginer, Ali Bulent Cengiz, Rae S. M. Yeung, Yasemin Ozsurekci, Seza Ozen

**Affiliations:** 1 Department of Pediatrics, Hacettepe University Faculty of Medicine, Ankara, Turkey; 2 Cell Biology Research Program, The Hospital for Sick Children Research Institute, Toronto, ON Canada; 3 Department of Pediatric Infectious Diseases, Hacettepe University Faculty of Medicine, Ankara, Turkey; 4 Department of Pediatric Intensive Care, Hacettepe University Faculty of Medicine, Ankara, Turkey; 5 Department of Pediatric Cardiology, Hacettepe University Faculty of Medicine, Ankara, Turkey; 6 Department of Pediatric Rheumatology, Hacettepe University Faculty of Medicine, Ankara, Turkey; 7 Division of Rheumatology, The Hospital for Sick Children, Toronto, ON Canada; 8 Departments of Paediatrics, Immunology and Institute of Medical Science, University of Toronto, Toronto, ON Canada

## Abstract

**Background:**

Multisystem Inflammatory Syndrome in Children (MIS-C) is a hyperinflammatory condition with multi-organ involvement, particularly affecting the cardiac and gastrointestinal systems. Although immunomodulatory therapy is standard, treatment approaches vary. This study aimed to evaluate treatment modalities in MIS-C such as methylprednisolone (MP), intravenous immunoglobulin (IVIG), anakinra and therapeutic plasma exchange (TPE) based on clinical severity and laboratory parameters in a prospectively followed cohort.

**Methods:**

A total of 125 MIS-C patients were included in the study and followed for at least 6 months after discharge. Patients were stratified by severity and treated with various immunomodulatory regimens, including IVIG+MP, IVIG+MP+anakinra, and IVIG+MP+anakinra+TPE.

**Results:**

Patients with mild disease and low inflammatory markers (median CRP 9 mg/dL, ferritin 192 µg/dL) received IVIG+MP. Those with higher inflammation (CRP 20–24 mg/dL, ferritin 308–846 µg/dL) without cardio-pulmonary support were treated with IVIG+low-dose-MP+anakinra. Patients with shock, macrophage activation syndrome, or bicytopenia received IVIG+high-dose-MP+anakinra. TPE was added in cases requiring cardio-pulmonary support. Most were discharged without corticosteroids or anakinra; only 11% received a short outpatient prednisolone taper.

**Conclusion:**

The mid-term longitudinal assessment of MIS-C patients suggests that timely immunomodulatory therapies, guided by laboratory parameters, promote safe resolution of systemic inflammation and cardiac complications, and shorten treatment duration.

**Impact:**

- Demonstrates that short-term, biomarker-guided use of anakinra and corticosteroids effectively controls hyperinflammation in MIS-C.
- Highlights that prolonged corticosteroid therapy may not be necessary, even in severe cases.
- Provides evidence of early cardiac recovery, including resolution of CAAs, without post-discharge steroids.
- Supports a steroid-sparing treatment approach, reducing risks of long-term immunosuppression.
- May inform future MIS-C treatment guidelines by minimizing the need for escalation therapy, ECMO, and related complications.

## Introduction

Multisystem Inflammatory Syndrome in Children (MIS-C) was defined in April 2020 as a Kawasaki disease-like clinical syndrome associated with post-SARS-CoV-2 infection.^1–4^ Centers for Disease Control and Prevention^3^ and World Health Organization^4^ released different but similar diagnostic criteria, emphasizing cardiac involvement, hyperinflammation and Kawasaki disease (KD) -like features and the need for excluding sepsis, staphylococcal toxic shock syndrome and KD in May 2020. Clinicians from around the world initially used their experience in KD for managing MIS-C empirically until the guidelines were established, since MIS-C and KD had similar manifestations, including fever, rash, conjunctival hyperemia, mucosal changes, lymphoadenopathy, extremity changes, gastrointestinal and cardiac involvement. However, hemodynamic instability in MIS-C required aggressive immunomodulatory therapies and intensive care modalities.^2,5,6^

In fact, uncontrolled hyperinflammation in patients with MIS-C was also leading to major complications such as shock and multiorgan failure. Thus, pediatric rheumatologists also used their experience in macrophage activation syndrome (MAS), by escalating therapy with anakinra and methylprednisolone,^7^ to control the cytokine storm in patients with MIS-C.

The lack of evidence-based randomized controlled studies in MIS-C management involving acute and subacute phase, as well as concerns about overlapping characteristics between Kawasaki disease, MAS, and staphylococcal toxic shock syndrome like shock and coronary artery abnormalities (CAAs), led to some variation in approaches for treating hyperinflammation and its complications with immunomodulatory therapies during hospitalization. The dose and duration of corticosteroids also varied considerably according to the clinical practice of the caring center.

Our aim was to assess our treatment regime in the light of the clinical characteristics and laboratory investigations in a prospectively followed (6 months) cohort. We also aimed to characterize the recovery of laboratory values with day-by-day monitoring to analyze accuracy and the outcomes of our stratified treatment approach, with titration of the corticosteroid and anakinra administration.

## Methods

### Design

Children who were hospitalized with the diagnosis of MIS-C between August 2020 and January 2023, and were followed for a minimum of 6-months at Hacettepe University, Department of Pediatrics were included in this study. The study was conducted according to the Helsinki Declaration’s ethical principles and Ethical Approval was received from the local ethics committee of Hacettepe University with the reference number GO22/172, which granted a waiver of informed consent.

### Cohort

**Fig. 1:**
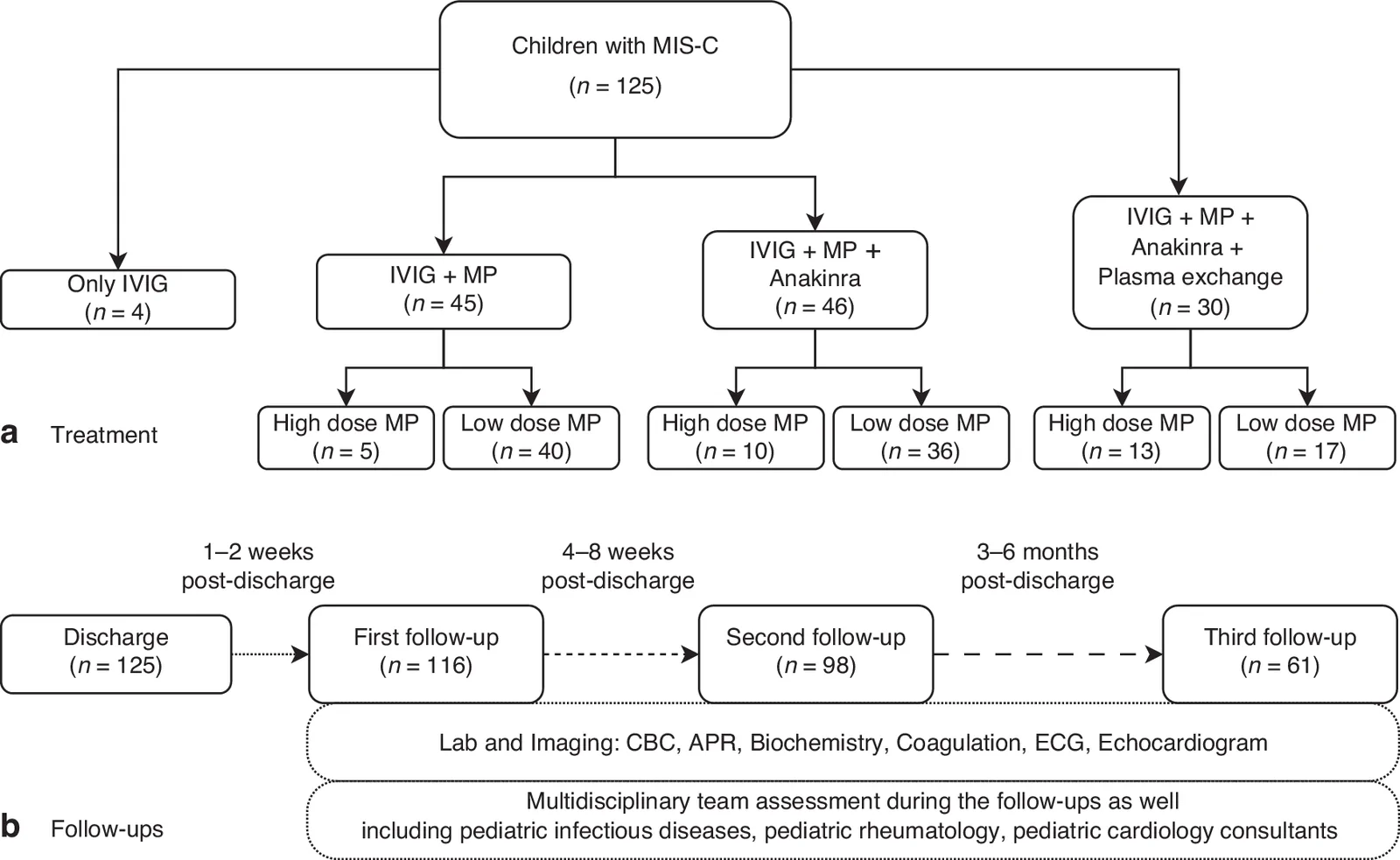
Treatment-based study flow and follow-up after discharge in MIS-C. **a** The flowchart of MIS-C patients who received four main immunomodulatory treatments including IVIG, IVIG+MP, IVIG+MP + Anakinra, IVIG+MP + Anakinra + Plasma Exchange. **b** Longitudinal follow-up plan in patients with MIS-C.

One hundred thirty-seven children with clinical suspicion of MIS-C were investigated. Twelve children were excluded: four with underlying rheumatologic or immunological diseases complicating the diagnosis, three with ‘classic’ KD meeting diagnostic criteria of the American Heart Association (AHA) for KD^8^ and had no history or existence of SARS-CoV-2 infection confirmed by polymerase chain reaction (PCR), Rapid Antigen Test, or SARS-CoV-2 ELISA IgG, two diagnosed with brucellosis, two with COVID-19/severe pneumonia, and one treated with anti-IL-6. In total 125 patients with MIS-C diagnosis under the age of 18 were included in this study (Fig. 1). Among them, 123 patients met the World Health Organization^4^ criteria for MIS-C upon admission, while two met the criteria of other organizations such as the Centers for Disease Control and Prevention (CDC)^3^ and the Canadian Paediatric Surveillance Program (CPSP), separately. Various associations’ criteria for MIS-C and Paediatric Inflammatory Multisystem Syndrome were evaluated in our cohort based on their presentation on admission and depicted in the Venn diagram (Supplementary Fig. S1). The treatments and follow-ups were planned with a team including specialists from the departments of pediatric infectious diseases, pediatric rheumatology, pediatric intensive care unit, and pediatric cardiology.

Infections with SARS-CoV-2 have been confirmed with PCR of nasopharyngeal samples, serology via ELISA, or rapid antigen detection kits.

The data of hospitalized patients involved the demographic and clinical features including symptoms, laboratory investigations, echocardiographic assessment, intensive care applications, and treatments.

### Definitions

The definitions of some clinical findings and organ involvement are reviewed in Supplementary Table S1.

### MIS-C phenotypes

i. *KD-like presentation:* The diagnostic criteria for KD from AHA defined the complete KD with fever >5 days and at least 4 of 5 following clinical symptoms: conjunctivitis, rash, oro-mucosal changes, extremity changes and cervical lymphadenopathy. In the case of having 2 or 3 criteria with CRP > 3 g/dL and/or ESR > 40 mm/h, to diagnose incomplete KD, positive echocardiography (ECHO) findings or at least 3 lab features including anemia for age, leukocytosis (>15 × 10^9^/L), thrombocytosis > 450 × 10^9^/L after 7 days of fever, hypoalbuminemia (<30 g/L), elevated ALT, pyuria (leukocyte  > 10/hpf) were used.^8^
ii. *Shock:* Age-dependent hypotension with impaired perfusion required inotropic support or fluid resuscitation.
iii. *Fever and inflammation (F&I):* Patients presenting with fever and hyperinflammation and positive SARS-CoV-2 IgG ELISA, but did not meet the incomplete KD criteria or shock.^2^

### Treatment approach

The management of MIS-C in our center was based on a structured algorithm that combined escalation and de-escalation strategies, determined by disease severity, clinical course, and laboratory findings. On admission, patients were evaluated for hyperinflammatory status (elevated CRP and ferritin), cytopenias (low platelet and lymphocyte counts), presence of MAS, the number of organ systems involved, cardiac dysfunction assessed by laboratory markers and echocardiography, and the need for cardio-pulmonary support. According to this assessment, initial therapy consisted of intravenous immunoglobulin (IVIG) alone for mild disease; IVIG with low-dose methylprednisolone (MP) for moderate disease without shock; and IVIG with low-dose MP and subcutaneous anakinra for moderate-to-severe disease with multi-organ involvement or cardiac dysfunction.

Escalation of treatment was undertaken in patients who showed no improvement within 12–24 h of initial therapy, as evidenced by persistent fever and abdominal pain, hypotension, less than an ~20% daily reduction in CRP, or failure of platelet and lymphocyte counts to recover. Escalation measures included increasing MP and increasing the dose of anakinra. In refractory cases with ongoing shock or hyperinflammation despite these steps, therapeutic plasma exchange (TPE) was considered, guided by daily echocardiographic evaluation and cardiac biomarker monitoring.

De-escalation was initiated once predefined response criteria were met: resolution of fever, abdominal pain and hypotension without inotropic support, recovery of platelet and lymphocyte counts and at least an ~20% daily fall in CRP. MP was tapered gradually, anakinra was reduced or stopped, and patients were switched to low-dose oral prednisolone, with most discharged without ongoing MP. Acetylsalicylic acid (ASA) was usually stopped after 6 weeks in the absence of coronary artery involvement (Supplementary Fig. S2).

### Follow-up

Following discharge, all patients were followed as per protocol and called for their first follow-up within 1 week, for their second visit within 1–4 week, and for the third follow-up within 3–6 months. One hundred sixteen, 98 and 61 patients attended first, second and third follow-ups, respectively. The consultants of the same multidisciplinary team followed these patients after the discharge at the same hospital (Fig. 1).

### Statistical analyses

Descriptive statistics were used for variables including patients’ clinical features, disease management, and laboratory values. Continuous variables were presented as median with interquartile range (IQR), and categorical variables were presented as frequency. For categorical variables, Fisher exact test or chi-square test of independence was used as appropriate. In the assessment of continuous variables Kruskal–Wallis and Mann–Whitney U test was used. For longitudinal data spanning admission, discharge, and follow-ups, linear mixed model analyzes were conducted using the ‘*lme4’* package. Twenty-four clinical features and laboratory values were included as the dependent variable separately, with time point and patient included as fixed and random effects, respectively. Tukey method was used to correct for multiple pairwise comparisons between time points. Conditional inference classification decision tree models using the R *‘party’* package and the *‘ctree’* algorithm,^9^ which recursively partitioned the data to identify optimal splitting variables and thresholds, which recursively partitions the data to identify optimal splitting variables and thresholds. A wide range of admission laboratory and clinical parameters were included: triglycerides, CRP, ferritin, procalcitonin, INR, LDH, albumin, troponin, brain natriuretic peptide (BNP), platelet count, absolute lymphocyte count (ALC), white blood cell count (WBC), hemoglobin, sodium, fibrinogen, IL-6, and clinical features such as pericardial effusion, valve insufficiency, shock, respiratory support, MAS, bicytopenia, cardiac involvement, and non-responsiveness to initial therapy. The algorithm determined optimal split variables and cut-off values based on statistical significance, rather than pre-specified clinical thresholds, to maximize separation between treatment groups. All analyzes and visualizations were performed using R, version 4.0.2 (R Foundation for Statistical Computing, Vienna, Austria).

## Results

All patients were Turkish and Caucasian and younger than 18 years of age. In patients with fever, inflammation, and KD-like features (*n* = 57), median age was 6, whereas patients with shock (*n* = 47) were significantly older (*p* value = 0.025 and 0.001) (Table 1). While 120 of 125 patients had positive SARS-CoV-2 IgG ELISA (>1.1), and 103 of 104 patients had reactivity on rapid antigen test, 7 of 121 patients had SARS-CoV-2 PCR positivity on admission. Patients with household contacts or who needed urgent medication or intensive care were tested with Rapid antigen test until ELISA results showed SARS-CoV-2 infection or exposure.

**Table 1:** Clinical features, laboratory parameters, and echocardiographic evaluation of patients with MIS-C stratified by MIS-C phenotypes.

Characteristic	Overall, *N* = 125^a^	Fever and Inflammation, *N* = 21^a^	KD, *N* = 57^a^	Shock, *N* = 47^a^	*p*-value
Age (Year)	8 (4–12)	6 (3–11)	6 (3–10)	10 (8–14)	<0.001^b^
Gender (M)	78 (62%)	16 (76%)	36 (63%)	26 (55%)	0.3^c^
Presentation					
Fever	5 (4–7)	4 (3–6)	5 (4–7)	5 (4–7)	0.024^b^
Number of KD features	3 (2–4)	2 (1–3)	3 (3–4)	3 (2–4)	<0.001^b^
Conjunctivitis	103 (82%)	14 (67%)	49 (86%)	40 (85%)	0.14^d^
Rash	79 (63%)	10 (48%)	44 (77%)	25 (53%)	0.011^c^
Oromucosal changes	89 (71%)	10 (48%)	50 (88%)	29 (62%)	<0.001^c^
Extremity changes	22 (18%)	0 (0%)	11 (19%)	11 (23%)	0.034^d^
Cervical lymphadenopathy	59 (47%)	6 (29%)	35 (61%)	18 (38%)	0.011^c^
Gastrointestinal involvement	102 (82%)	16 (76%)	44 (77%)	42 (89%)	0.2^d^
Abdominal pain	82 (66%)	11 (52%)	32 (56%)	39 (83%)	0.006^c^
Vomiting	69 (55%)	13 (62%)	25 (44%)	31 (66%)	0.062^c^
Diarrhea	61 (49%)	10 (48%)	22 (39%)	29 (62%)	0.063^c^
CNS involvement	34 (27%)	4 (19%)	11 (19%)	19 (40%)	0.036^c^
HA	24/124 (19%)	2/20 (10%)	10/57 (18%)	12/47 (26%)	0.3^d^
AMS	13/124 (10%)	3/20 (15%)	2/57 (3.5%)	8/47 (17%)	0.043^d^
Others	8/124 (6.5%)	2/20 (10%)	1/57 (1.8%)	5/47 (11%)	0.10^d^
Renal involvement	58/113 (51%)	8/20 (40%)	21/49 (43%)	29/44 (66%)	0.046^c^
Proteinuria	39/109 (36%)	6/20 (30%)	16/49 (33%)	17/40 (43%)	0.5^c^
Hematuria	15/109 (14%)	3/20 (15%)	5/49 (10%)	7/40 (18%)	0.6^d^
AKI	20/123 (16%)	0/20 (0%)	3/57 (5.3%)	17/46 (37%)	<0.001^d^
MAS	28 (22%)	1 (4.8%)	8 (14%)	19 (40%)	<0.001^d^
Lab values					
Hemoglobin (102–127 g/L)	114 (105–123)	122 (113–129)	114 (107–123)	109 (98–118)	0.003^b^
White blood cell (4.0–13.8 × 10^9^/L)	10.1 (6.6–14.7)	10.5 (7.5–12.5)	8.9 (6.3–13.4)	11.7 (7.3–17.0)	0.2^b^
Absolute Neutrophil Count (1.6–8.3 × 10^9^/L)	7.3 (4.8–12.0)	7.2 (4.8–9.8)	6.8 (4.3–10.5)	9.8 (6.0–15.0)	0.033^b^
Absolute Lymphocyte Count (1.3–5.8 × 10^9^/L)	1.1 (0.7–1.7)	1.1 (0.7–2.5)	1.2 (0.8–1.8)	0.9 (0.6–1.3)	0.006^b^
Platelet count (189–394 × 10^9^/L)	181 (119–292)	204 (163–292)	199 (110–342)	154 (106–202)	0.013^b^
C-reactive protein (<0.5 mg/dL)	18.1 (10.2–24.6)	13.5 (8.3–23.3)	13.2 (8.6–20.6)	22.6 (18.1–28.4)	<0.001^b^
Ferritin (5.3–99.9 ug/L)	345.0 (165.7–678.6)	182.2 (98.5–308.4)	266.1 (136.8–456.6)	688.1 (374.8–1151.0)	<0.001^b^
ESR (mm/h)	40 (22–53)	29 (20–48)	41 (22–53)	37 (23–58)	0.5^b^
IL-6 (<6.4 pg/mL)	75.0 (29.0–159.7)	86.6 (37.3–169.8)	53.8 (25.8–143.8)	77.0 (41.9–206.8)	0.5^b^
Procalcitonin (<0.1 ng/mL)	4.5 (1.5–14.5)	1.4 (0.9–11.7)	2.2 (0.8–6.1)	11.7 (6.0–27.4)	<0.001^b^
Sodium (136–146 meq/L)	134 (132–136)	135 (133–137)	134 (133–136)	134 (131–136)	0.5^b^
Albumin (35–47 g/L)	32.1 (27.6–36.2)	34.1 (32.3–38.6)	33.7 (30.9–38.3)	27.7 (25.8–30.5)	<0.001^b^
Creatinine (0.26–0.77 mg/dL)	0.4 (0.3–0.6)	0.3 (0.3–0.5)	0.4 (0.3–0.5)	0.6 (0.4–0.8)	<0.001^c^
ALT (<39 U/L)	31 (18–65)	18 (14–43)	32 (17–60)	31 (24–73)	0.12^b^
AST (<56 U/L)	36 (26–56)	35 (26–41)	36 (25–50)	38 (28–77)	0.4^b^
LDH (110–295 U/L)	312 (256–370)	264 (231–335)	315 (256–370)	316 (288–394)	0.034^b^
Triglycerides (<150 mg/dL)	189 (121–268)	123 (70–214)	173 (117–251)	240 (170–326)	0.002^b^
INR (0.8–1.2)	1.2 (1.1–1.3)	1.2 (1.1–1.3)	1.2 (1.1–1.3)	1.2 (1.2–1.4)	0.009^b^
PTT (24–36 s)	26 (23–29)	24 (22–26)	25 (22–28)	28 (26–30)	<0.001^b^
Fibrinogen (180–350 mg/dL)	486 (406–663)	516 (450–633)	480 (396–639)	485 (386–672)	0.5^b^
D-dimer (<0.55 mg/L)	3.7 (1.9–6.8)	1.8 (1.1–4.5)	3.3 (2.0–7.8)	4.7 (3.0–7.0)	0.008^b^
Troponin (8.4–18.3 ng/L)	24.0 (6.1–133.6)	8.0 (2.4–28.6)	11.6 (3.8–38.1)	66.3 (26.0–464.1)	<0.001^b^
BNP (<100 pg/mL)	195.2 (62.3–962.4)	82.1 (34.0–201.0)	107.6 (48.9–333.0)	1,069.0 (183.0–2222.7)	<0.001^b^
Microbiologic					
SARS-CoV-2 PCR positive	7/121 (5.8%)	1/21 (4.8%)	5/56 (8.9%)	1/44 (2.3%)	0.3^d^
Rapid antigen test (Reactive)	103/104 (99%)	17/17 (100%)	46/46 (100%)	40/41 (98%)	0.6^d^
Covid-19 ELISA IgG (positive>1.1)	3.6 (2.4–7.5)	4.3 (2.9–6.5)	3.7 (2.4–8.0)	3.0 (1.9–6.0)	0.3^b^
Co-infection with another viruses	7 (5.6%)	2 (9.5%)	4 (7.0%)	1 (2.1%)	0.3^d^
Echocardiography					
EF (%)	64.5 (56.0–70.3)	65.0 (64.0–70.3)	68.5 (62.0–73.8)	54.5 (50.0–63.0)	<0.001^b^
EF < 55%	26 (21%)	0 (0%)	1 (1.8%)	25 (53%)	<0.001^d^
Valve insufficiency	55 (44%)	1 (4.8%)	20 (35%)	34 (72%)	<0.001^c^
Pericardial effusion	21 (17%)	0 (0%)	5 (8.8%)	16 (34%)	<0.001^d^
Cardiac abnormality	92 (74%)	11 (52%)	36 (63%)	47 (100%)	<0.001^c^
Abnormal Z-score any time (>2.0)	9 (7.2%)	1 (4.8%)	7 (12%)	1 (2.1%)	NA
Abnormal Z-score during hospitalization (>2.0)	7 (5.6%)	1 (4.8%)	5 (8.8%)	1 (2.1%)	NA
Abnormal Z-score at first follow up (>2.0)	2/86 (2.3%)	0/13 (0%)	2/39 (5.1%)	0/34 (0%)	NA
Abnormal Z-score at second follow up (>2.0)	64/64 (100%)	12/12 (100%)	30/30 (100%)	22/22 (100%)	NA
Abnormal Z-score at third follow up (>2.0)	0/46 (0%)	0 / 6 (0%)	0/18 (0%)	0/22 (0%)	NA

*CNS* central nervous system, *HA* headache, *AMS* altered mental status, *AKI* Acute kidney injury, *MAS* macrophage activation syndrome, *ALT* alanine aminotransferase, *AST* aspartate aminotransferase, *LDH* lactate dehydrogenase, *PTT* partial thromboplastin time, *BNP* B-type natriuretic peptide.

^a^*n* (%); Median (IQR); *n*/*N* (%).

^b^Kruskal–Wallis rank sum test.

^c^Pearson’s Chi-squared test.

^d^Fisher’s exact test.

### Admission

#### a. Clinical features

Among 125 cases, most common symptoms following fever were conjunctivitis (82%) and mucosal changes (71%) and abdominal pain (66%). Central nervous system (CNS), renal, and cardiac involvement were observed in one, two, and three quarters of patients, respectively; gastrointestinal system (GI) involvement was found in 82% of patients. In total, 120 out of 125 patients had involvement of two or more organs including cardiac, pulmonary, GI, renal, CNS and dermatologic.

In total, 47 presented with (38%) shock, 57 (46%) with KD, and 21 (17%) with fever and inflammation phenotypes. Abdominal pain was present significantly in shock (83%, *p* = 0.017 and *p* = 0.047) group more than KD (56%) and F&I (52%). In 37% and 40% of shock patients, acute kidney injury and MAS were diagnosed, respectively, with significant differences (Table 1).

#### *b*. Lab values

All patients at admission had increased at least 5 laboratory evidence of inflammation in accordance with CDC criteria.^3^ Patients with shock had significantly decreased hemoglobin, ALC, platelet, and albumin; and elevated CRP, ferritin, procalcitonin, triglyceride, INR, aPTT, troponin and BNP level at admission. There was no significant difference in WBC count and ESR, IL-6, Na, and fibrinogen levels among phenotypes (Table 1).

#### *c*. Echocardiography^10^^10^

ECHO was performed for each patient at least one time during hospitalization. Repeated ECHO was performed for patient who required inotropic support with shock phenotypes, thus ejection fraction (EF) in half of these cases were greater than 55%. Three-quarters of the shock patients developed valve insufficiency, and pericardial effusion was in one-third. From admission to last follow-up, A total of nine patients (7.2%) with CAAs (Z-score >2 in Boston criteria) were found, two of whom were aneurysm (Z-score > 2.5). Seven of these CAAs resolved within the 6 months. One patient presented with CAAs during hospitalization but was lost to follow-up after discharge, while another was diagnosed with coronary dilation at the first follow-up but did not attend subsequent evaluations (Table 1 and Supplementary Table S4).

#### d. Management

Based on the clinical severity of patients with MIS-C, as assessed by hyperinflammatory status with high CRP and ferritin levels, low platelet and lymphocyte counts, presence of MAS, number of organ involvements, the spectrum of cardiac dysfunction with myocarditis, and the need for cardio/pulmonary support, treatment initiation followed immediately after diagnosis. The initial treatments for timely administration were IVIG, IVIG + low-dose methylprednisolone (MP),^7^ or IVIG + low-dose MP + anakinra. Escalation performed for patients not responding to the initial treatment within 24 h, especially after 12 h of IVIG administration. Clinical response with laboratory investigation guided decisions for escalation as well. In the absence of clinical improvement, involving parameters like fever, hypotension, and laboratory values, low-dose MP was escalated to pulse, and/or Anakinra was added. The TPE modality was performed in 64% of patients with shock who were unresponsive to IVIG+MP + anakinra within 24–48 h as the last step of escalation (Supplementary Fig. S2).

All patients received IVIG infusion (2 gr/kg/d [max 100 gr], 12 h). Almost all patients, apart from 4, received IVIG+MP^7^ during admission, either in low MP (1–4 mg/kg/d) or high dose MP (5–30 mg/kg/d) depending on the severity of their condition. Basically, patients with MIS-C received one of the four treatment regimens: IVIG (*n* = 4), IVIG+MP (*n* = 45), IVIG+MP + anakinra (*n* = 46), and IVIG+MP + anakinra + TPE (*n* = 30) (Fig. 1). The clinical characteristics, laboratory features and accompanying management details of those who received these immunomodulatory treatment regimens are reviewed in Supplementary Table S2.

**Fig. 2:**
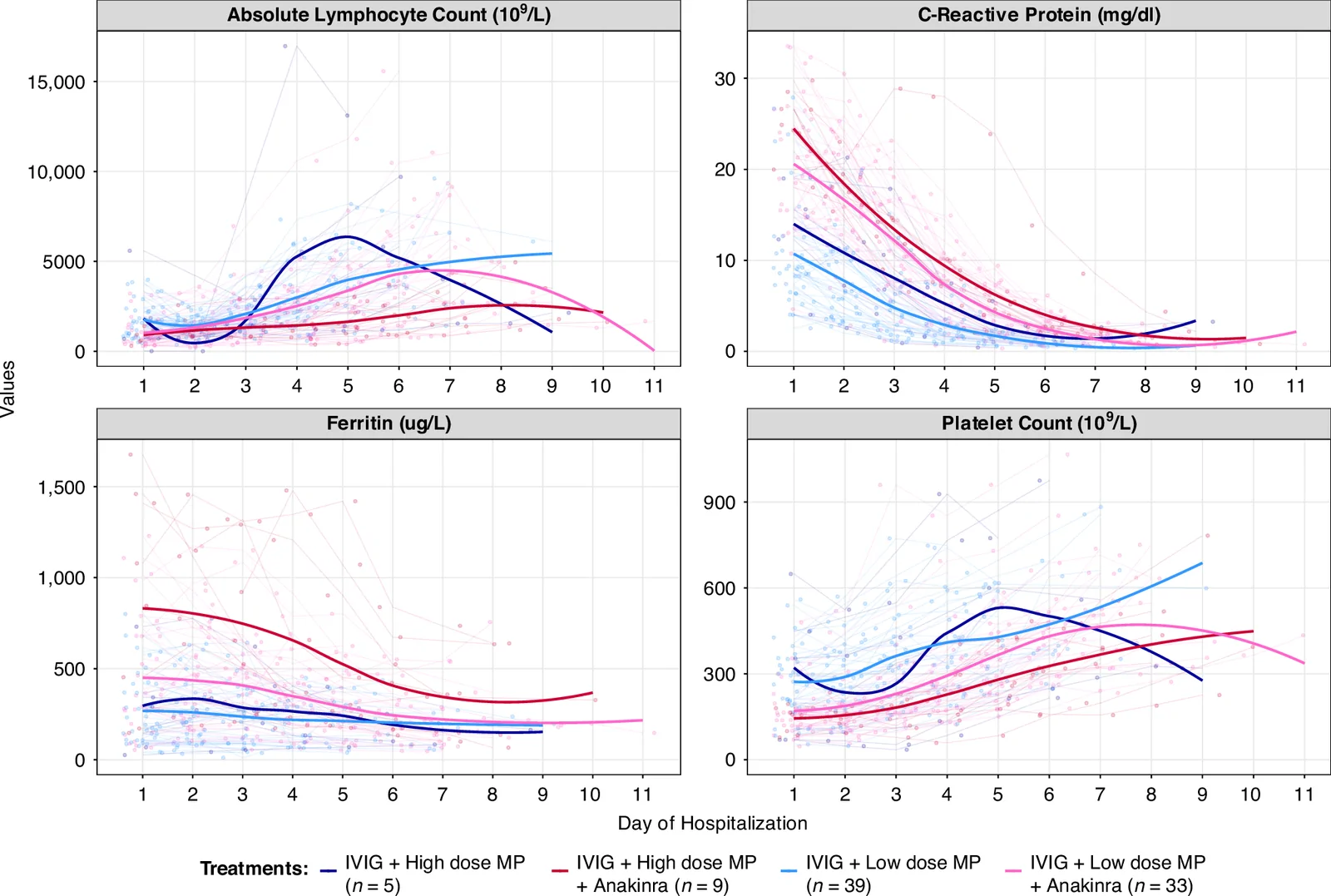
Daily trends in inflammatory and hematologic parameters in MIS-C patients receiving different immunomodulatory regimens. Serial measurements of absolute lymphocyte count, C-reactive protein (CRP), ferritin, and platelet count are shown from hospital admission through day 11, stratified by treatment groups: IVIG + low-dose methylprednisolone (MP) (*n* = 39), IVIG + high-dose MP (*n* = 5), IVIG + low-dose MP + anakinra (*n* = 33), and IVIG + high-dose MP + anakinra (*n* = 9). Lines represent smoothed trajectories, and points indicate individual patient values. Patients were excluded if they had incomplete daily laboratory records, missing baseline measurements, treatment regimens outside the four predefined groups, or were discharged early on oral corticosteroids, to ensure consistency in temporal comparisons across cohorts.

In 61% of patients with MIS-C, anakinra treatment (1–8 mg/kg/day) was administered subcutaneously for 4.4 days and was discontinued at discharge in all. The initial dosage regimen for anakinra typically was 4 mg/kg/day. In patients undergoing treatment with IVIG + low dose MP + anakinra, the initial dosage of anakinra was reduced from 4 to 2 mg/kg/day over a mean duration of 2.4 days, with an IQR of 2–3. The overall mean duration of anakinra administration in those who received IVIG + low dose MP + Anakinra was 4.4 days (Supplementary Fig. S3). This dose adjustment was predicated on the sustained downtrend in CRP, and uptrend in ALC and platelet levels noted during daily monitoring (Fig. 2). Similarly, in patients who received IVIG + high dose MP + anakinra, anakinra was reduced from 4 to 2 mg/kg/day within a mean duration of 2.8 days (IQR 2–3) and used for 4.6 days (IQR 4–5). In patients treated with IVIG + low-dose MP, IVMP was used at the doses of 0.5–4 mg/kg/day in median 5 days, and the average daily percentage decrease of CRP between administration and discharge under titration was 23.6% (Supplementary Fig. S4). MP was discontinued at discharge in all cases except for 14 cases.

**Fig. 3:**
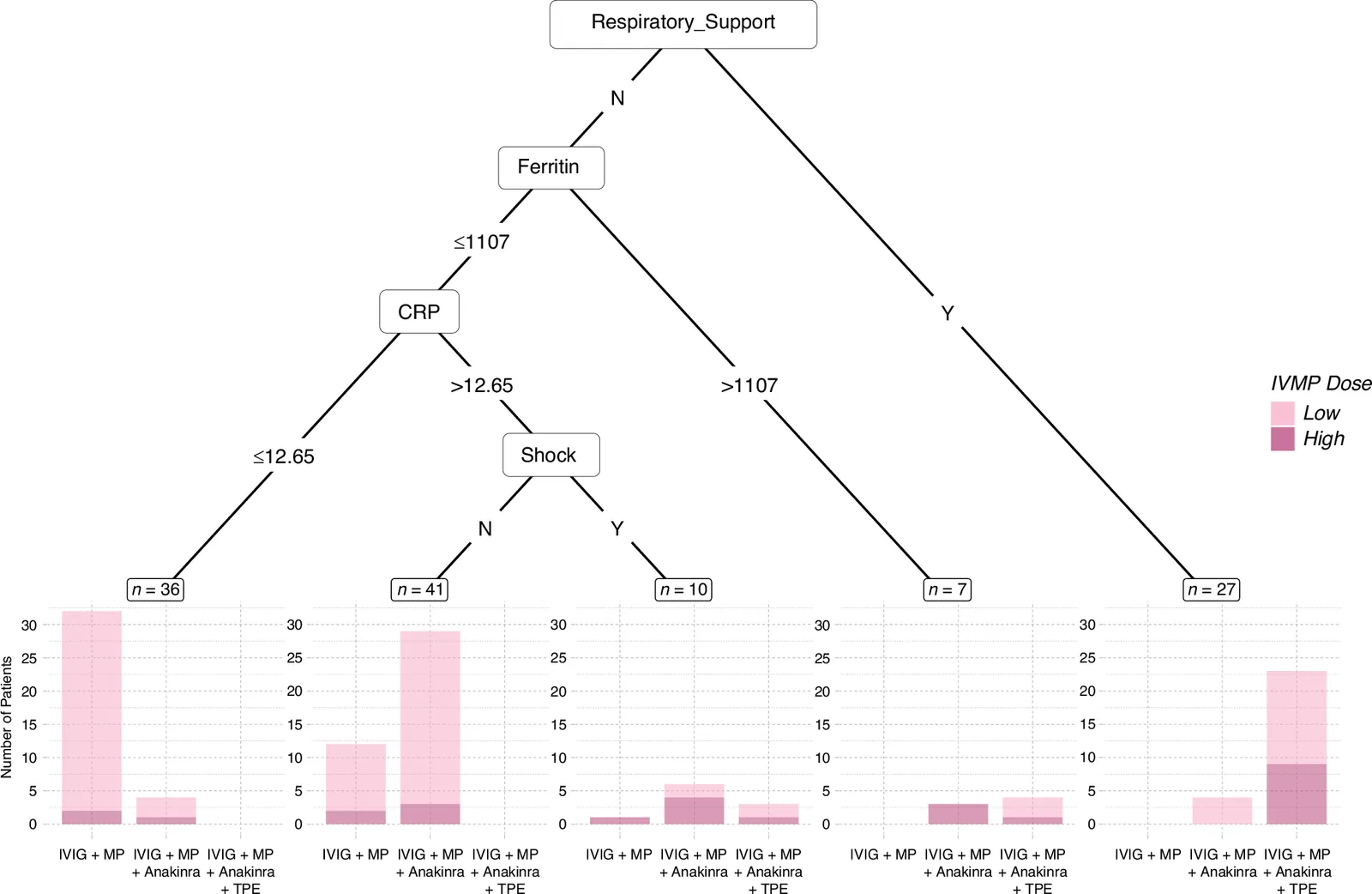
Classification and regression tree (CART) analysis showing treatment choices among three main immunomodulatory modalities: IVIG+MP, IVIG+MP + Anakinra, and IVIG+MP + Anakinra+TPE. “Y” (Yes) and “N” (No) indicate whether the preceding clinical criterion was met (e.g., respiratory support: Y = required, N = not required). Each node represents a clinical criterion guiding treatment, determined through binary recursive partitioning. Laboratory cut-off values for CRP (>12.65 mg/dL) and ferritin (>1107 µg/L) were determined automatically by the CART algorithm, as described in the Methods, to maximize separation between treatment groups. Bars show the number of patients receiving each treatment within terminal nodes, stratified by low- versus high-dose methylprednisolone (MP). TPE- or Anakinra-based therapies were generally used in patients requiring respiratory support, whereas TPE was also used in those without respiratory support but with ferritin >1107 µg/L and MAS. Patients without respiratory support, ferritin ≤1107 µg/L, and CRP ≤ 12.65 mg/dL most often received IVIG + low-dose MP. Patients with CRP > 12.65 mg/dL without shock were typically managed with IVIG + low-dose MP plus Anakinra.

In the sub-analysis of patients whose inflammation was controlled within 48 h, mildly affected patients with CRP levels around 9.1 mg/dL were treated with IVIG + low-dose MP. Patients with a median CRP level of 20.4 mg/dL.2 and/or evidence of cardiac involvement and/or bicytopenia received IVIG + low dose MP + anakinra. In contrast, patients presenting with significantly elevated CRP levels (median 23.4 mg/dL), and median ferritin levels of 852 μg/L were treated with IVIG + high dose MP + anakinra (Fig. 2 and Supplementary Fig. S5). TPE were combined with these treatments if there were clear signs of shock and/or need of respiratory support and/or presence of MAS (Fig. 3 and Supplementary Fig. S6). The CRP and ferritin level, and lymphocyte and thrombocyte count at Day 4 of these patients showed no significant differences between those who did not receive TPE (Supplementary Fig. S7) and between those who received TPE with low-dose MP and high-dose MP (Supplementary Fig. S8). Moreover, CRP levels declined to baseline in all treatment groups, whereas ferritin normalization occurred more gradually, most often after the first or second follow-up. Platelet counts returned to the normal range during hospitalization, and thrombocytosis, commonly seen in Kawasaki disease, was not observed in our MIS-C cohort.

Anticoagulation and/or antiplatelet treatment were a part of standard management. Of all, 119 (95%) patients received enoxaparin (0.5–1 mg/kg/dose every 12 h) and 109 (87%) were discharged with ASA (2–3 mg/kg/d) treatment (Table 2). Thrombosis or hemorrhage was not observed in patients.

**Table 2:** Management of patients with MIS-C stratified by MIS-C phenotypes.

Characteristic	Overall, *N* = 125^a^	Fever and Inflammation, *N* = 21^a^	KD, *N* = 57^a^	Shock, *N* = 47^a^	*p*-value
Management					
Length of hospitalization	6.0 (5.0–7.0)	5.0 (4.0–6.0)	5.0 (4.0–6.0)	7.0 (6.0–8.5)	<0.001^b^
PICU	34 (27%)	1 (4.8%)	1 (1.8%)	32 (68%)	<0.001^c^
Respiratory support	27 (22%)	1 (4.8%)	0 (0%)	26 (55%)	<0.001^d^
Max Respiratory support					0.6^d^
Nasal oxygen	9/27 (33%)	1/1 (100%)	0/0 (NA%)	8/26 (31%)	
NIMV	11/27 (41%)	0/1 (0%)	0/0 (NA%)	11/26 (42%)	
IMV	7/27 (26%)	0/1 (0%)	0/0 (NA%)	7/26 (27%)	
Inotropic agent	43 (34%)	0 (0%)	0 (0%)	43 (91%)	<0.001^c^
IVIG	125 (100%)	21 (100%)	57 (100%)	47 (100%)	
Second IVIG	7 (5.6%)	0 (0%)	0 (0%)	7 (15%)	0.002^d^
Immunomodulatory treatment					<0.001^d^
Only IVIG	4/125 (3.2%)	1/21 (4.8%)	3/57 (5.3%)	0 (0%)	
MP+IVIG	45/125 (36%)	11/21 (52%)	31/57 (57%)	3/47 (6.4%)	
MP+IVIG+ Anakinra	46/125 (37%)	9/21 (43%)	22/57 (41%)	15/47 (32%)	
MP+IVIG+ Anakinra + therapeutic plasma exchange	30/125 (21%)	0/21 (0%)	1/57 (1.8%)	29/47 (62%)	
IVMP	121 (97%)	20 (95%)	54 (95%)	47 (100%)	0.3^d^
IVMP dose					<0.001^d^
High dose (5–30 mg/kg/d)	29/121 (24%)	0/20 (0%)	7/54 (13%)	22/47 (47%)	
Low dose (1–4 mg/kg/d)	92/121 (76%)	20/20 (100%)	47/54 (87%)	25/47 (53%)	
Cumulative IVMP dose	11 (8–18)	8 (6–9)	12 (8–18)	15 (8–32)	0.001^b^
Duration IVMP (Days)	6 (5–7)	5 (4–5)	5 (4–6)	7 (5–9)	<0.001^b^
Oral prednisolone at discharge	14 (11%)	2 (9.5%)	7 (12%)	5 (11%)	>0.9^d^
Anakinra	76 (61%)	9 (43%)	23 (40%)	44 (94%)	<0.001^c^
Duration of Anakinra	4 (3–5)	3 (3–4)	4 (3–5)	5 (4–5)	0.070^b^
Plasma Exchange	31 (25%)	0 (0%)	1 (1.8%)	30 (64%)	<0.001^c^
Enoxaparin	119 (95%)	18 (86%)	55 (96%)	46 (98%)	0.14^d^
Acetylsalicylic acid	109 (87%)	19 (90%)	52 (91%)	38 (81%)	0.3^d^
Antibiotics	118 (94%)	20 (95%)	51 (89%)	47 (100%)	0.053^d^
Favipiravir	109 (87%)	17 (81%)	48 (84%)	44 (94%)	0.2^d^
Insulin	20 (16%)	0 (0%)	1 (1.8%)	19 (40%)	<0.001^d^
Any blood product	37 (30%)	1 (4.8%)	4 (7.0%)	32 (68%)	<0.001^c^
Albumin	28 (22%)	1 (4.8%)	2 (3.5%)	25 (53%)	<0.001^d^
Fresh frozen plasma	24 (19%)	0 (0%)	0 (0%)	24 (51%)	<0.001^d^
Transfusion					0.4^d^
Erythrocyte	8/10 (80%)	0/0	1/2 (50%)	7/8 (88%)	NA
Thrombocyte	2/10 (20%)	0/0	1/2 (50%)	1/8 (13%)	NA

*PICU* pediatric intensive care unit, *IMV* invasive mechanical ventilation, *NIMV* non-invasive mechanical ventilation.

^a^*n* (%); Median (IQR); *n*/*N* (%).

^b^Kruskal–Wallis rank sum test.

^c^Pearson’s Chi-squared test.

^d^Fisher’s exact test.

Patients with shock required 7 (6–8.5) days hospitalization, which was significantly longer than KD and F&I (*p* = 0.001). In the shock cohort, 43/47 (91%) required at least one inotropic agent, while intravenous fluid resuscitation stabilized hypotension for the remainder. One patient underwent extracorporeal membrane oxygenation (ECMO). Respiratory support was required in 55% of cases with shock. All patients survived (Table 2).

Following discharge, there were two patients (1.6%) who were readmitted due to abdominal pain with no fever or elevated CRP. Immunomodulatory therapy was not repeated for the two patients; only antibiotic therapy was administered and then they were discharged without complication. Interestingly, at the sixth week post-discharge, a girl with shock phenotype was diagnosed with seropositive ocular myasthenia gravis.^11^

#### e. Discharge and follow-ups

**Fig. 4:**
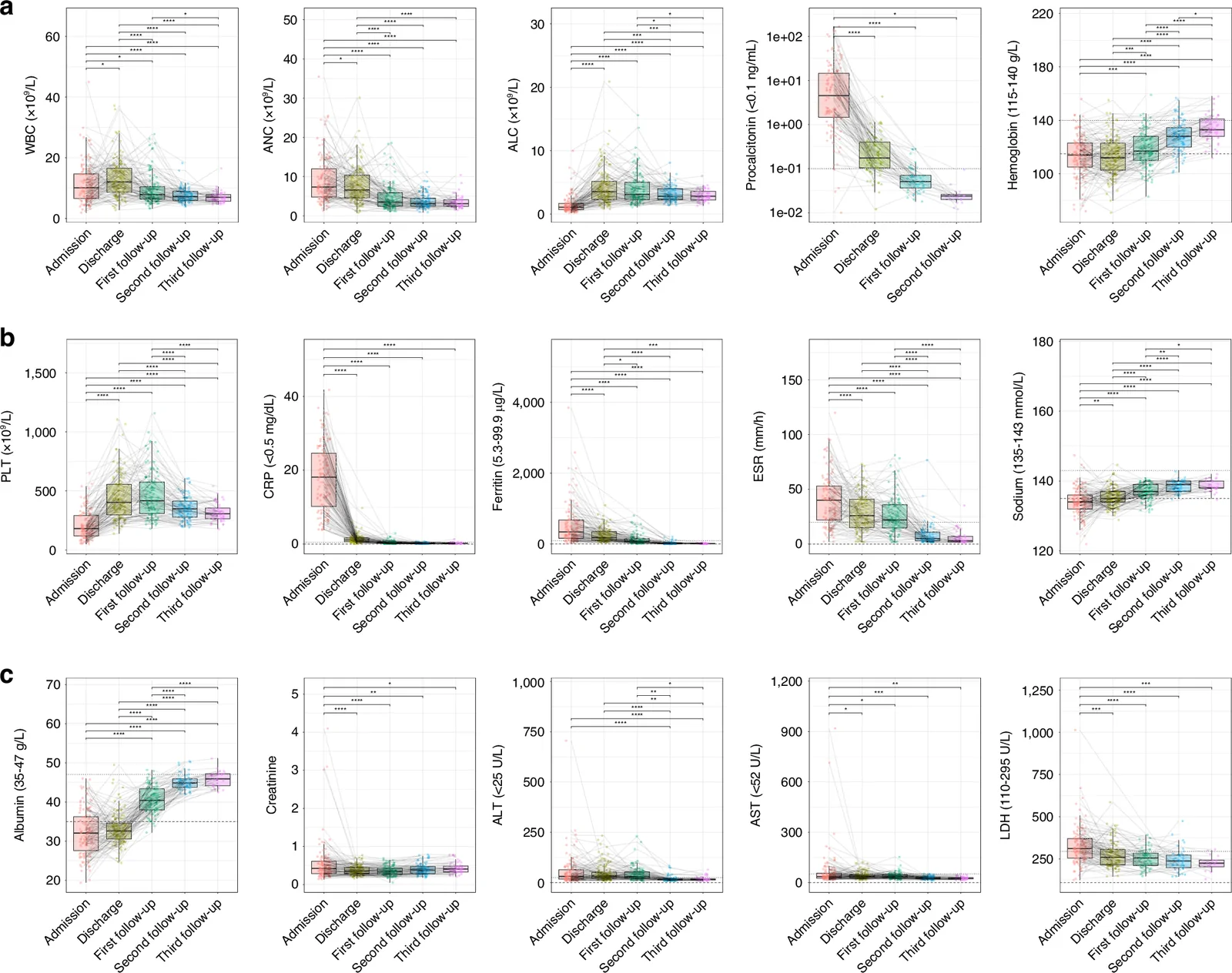

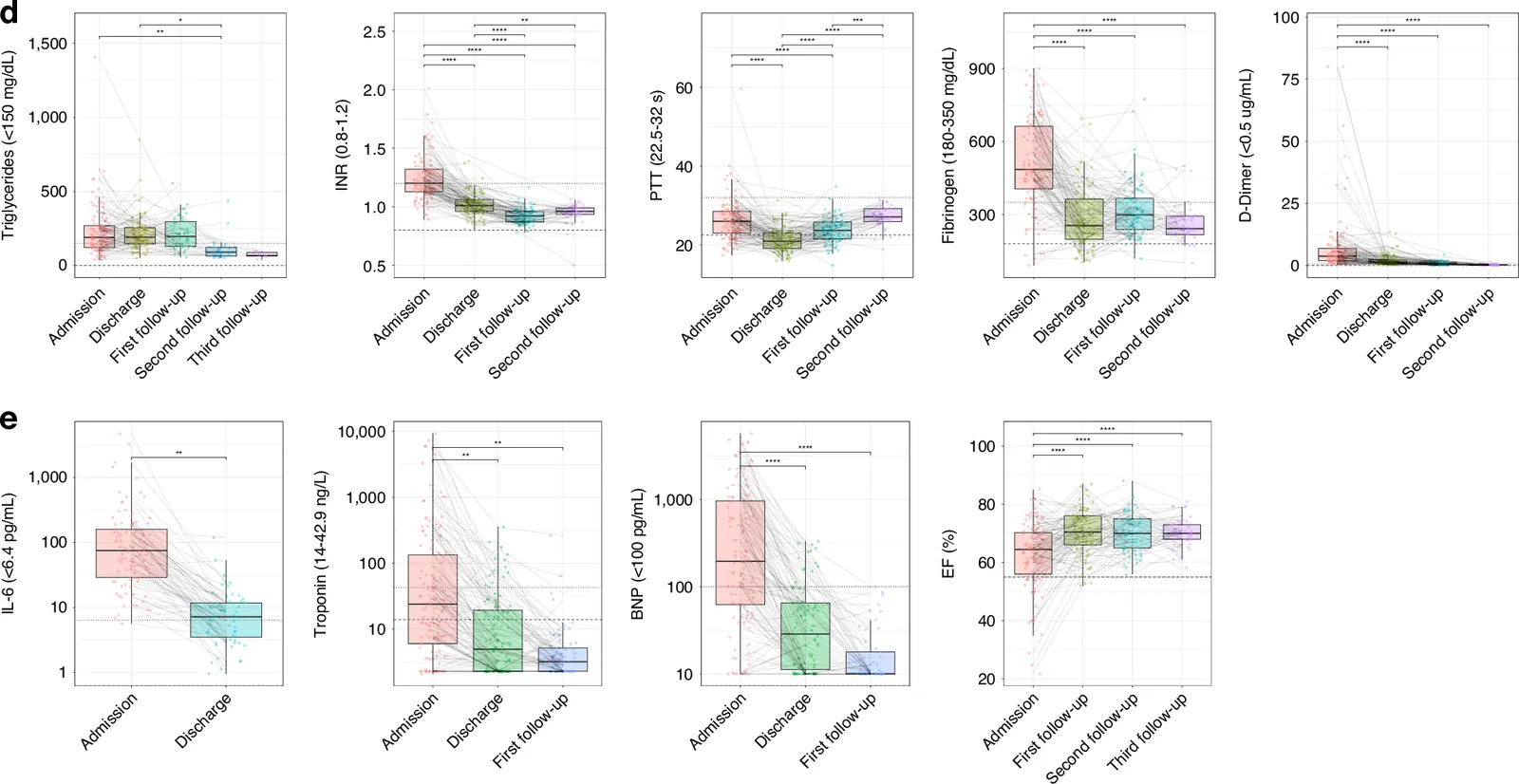
Longitudinal monitoring of laboratory markers with pairwise statistical comparisons. Serial measurements of inflammatory, hematologic, and biochemical parameters were obtained during hospitalization and follow-up visits in patients with MIS-C. Each panel displays changes over time for a specific marker, with individual patient trajectories (grey lines) overlaid on boxplots representing median and interquartile ranges at each time point. Data points represent repeated measurements per patient, connected by lines. Group differences at each time point were assessed using linear mixed-effects models with patient ID as a random effect, followed by -adjusted post-hoc pairwise comparisons via estimated marginal means. Statistical significance is indicated by asterisks: *p* ≤ 0.05 (*), *p* ≤ 0.01 (**), *p* ≤ 0.001 (***), and *p* ≤ 0.0001 (****). **a** Complete blood counts (white blood cells, absolute neutrophil count (ANC), absolute lymphocyte count (ALC), hemoglobin, platelet count). **b** Inflammation markers (procalcitonin, C-reactive protein (CRP), ferritin, erythrocyte sedimentation rate (ESR), interleukin-6 (IL-6)). **c**. Biochemical and hepatic parameters (sodium, albumin, creatinine, alanine aminotransferase (ALT), aspartate aminotransferase (AST)). **d** Metabolic and coagulation markers (lactate dehydrogenase (LDH), triglycerides, international normalized ratio (INR), partial thromboplastin time (PTT), fibrinogen). **e** Cardiac markers and D-dimer (D-dimer, troponin, B-type natriuretic peptide (BNP), ejection fraction by ECHO (EF)).

Length of hospitalization for F&I and KD like features was 6 (4–6) days while patients with shock had a median stay of 7 days. During the hospitalization, there was a significant increase observed in WBC, ALC, and platelet count, accompanied by a decrease in ANC. While BNP, troponin, fibrinogen returned to their normal range at discharge, CRP, procalcitonin and albumin normalized at the first follow-up; Ferritin, ESR, triglyceride, LDH and D-dimer returned to normal around the second follow-up. (Fig. 4 and Supplementary Table 3).

A total of 116 patients were admitted to at the first follow-up interval. Only 6 had high CRP (>1.0 mg/dl) ranging between 1.24 and 2.94 mg/dL without symptoms, and only one had rebound inflammation which was self-limited. The rest of the patients displayed a gradual normalization of CRP levels at discharge. Also, among these 6 patients, only one was prescribed a post-discharge steroid based on their CAAs risk factor.

Throughout the follow-up period, a total of nine patients developed CAAs. Two patients, both treated with anakinra-based therapies, exhibited aneurysms during hospitalization—one with a right coronary artery (RCA) Z-score of 3.13 at admission, and another with a left main coronary artery (LMCA) Z-score of 2.58, which resolved by the first follow-up. The remaining seven patients had Z-scores between 2 and 2.5; all but one received anakinra-based therapy, and none were discharged with corticosteroid treatment. Remarkably, CAAs resolution occurred in six of the seven patients, whereas one case who treated with IVIG+MP (LMCA Z-score: 1.7 and RCA Z-score: 2.1) failed to attend the first follow-up. In two cases who did not have CAAs at admission, but CAA developed at their first follow-up sessions on the 12th and 9th days, IVIG+MP had been administered. One patient demonstrated resolution at the second follow-up, whereas the other case (LMCA Z-score: 2.2 and RCA Z-score: 2.1) did not come to the scheduled second follow-up (Supplementary Table S4).

## Discussion

Despite of the short-term use of steroids, low cardiac complication of our cases might possibly be attributed to our management procedures that mainly include anakinra in addition to IVIG and MP. It has been reported that anakinra is efficacious in suppressing fever and markers of systemic inflammation, as well as preventing coronary artery dilatation in Kawasaki disease^12,13^. The use of anakinra as a first-line short-course monotherapy for patients with new-onset systemic juvenil idiopathic arthritis was also shown to be effective in terms of both short and long-term results.^14^ Anakinra was also used during the COVID-19 pandemic to restore pro-/anti-inflammatory balance in COVID-19 hyperinflammation.^15^ The use of anakinra in MIS-C was also based on efficacy studies in MAS, steroid-sparing properties, short half-life and recent research with the sufficient outcome data. Anakinra use in MIS-C was also reported with favorable outcomes in fever resolution and cardiac function, preventing persistent heart disease.^16–18^ Version 1^19^ of the American College of Rheumatology^20^ guideline in 2020 recommended IVIG alone for mild MIS-C cases or when there were contraindications to glucocorticoids, or IVIG + low-dose MP as first-line treatment for MIS-C. If patients receiving first-line treatment experienced persistent fevers and/or significant end-organ involvement, this was considered refractory disease, and therapy should be escalated with high-dose MP, anakinra, or infliximab. However, based on comparative studies, the recommendation for first-line treatment was updated in Version 3^21^ to IVIG in combination with glucocorticoids. Our cohort largely followed this approach prior to the publication of Version 3, with IVIG and glucocorticoids used as first-line therapy in most patients.

Anakinra was the preferred biologic in most pediatric centers.^16–18,22–27^ Because the presence of high percentage of severe cases in our cohort, anakinra was a reasonable drug choice in our center, as well. In the current cohort, anakinra was used in the patients with cardiac dysfunction together with more than two organ involvement particularly in which the need of respiratory support or presence of MAS or in those with refractory hyperinflammation. The improvement of cardiac findings of the patients was satisfactory. In addition, it was considered according to the findings of the present study that short-term anakinra treatment, which is started and tapered quickly allows us to monitor patients without seeing disease exacerbations during follow-up even though we use the steroids for a short time. Thus, anakinra-based treatments may have a potential to provide fast and safe improvement without complication, which mainly based on dose titration according to CRP and ALC.

Before the aforementioned guideline, the patients with MIS-C were managed with only IVIG or only MP.^19^ Even though Best Available Treatment Study (BATS) had found no significant differences in outcomes between those who received only IVIG, only glucocorticoid,^27^ or combination of them, only IVIG or glucocorticoid^27^ treatment necessitated more than 50% escalation therapy with GC or IVIG. Besides, combination of IVIG and MP was associated with faster recovery in cardiac depression and weaning from inotropic agents.^28,29^ On the other hand, it seems that CAAs prevalence during hospitalization and its resolution at discharge and follow-ups had no significant difference among these combinations. Most of these patients were discharged with continued use of corticosteroids in these series.^22,26,27,30,31^ Just like in the literature, we preferred to use the combination of IVIG and MP together in our patients, but unlike the literature, we did not use steroids after discharge. The patients with CAA at admission and treated with anakinra-based therapies demonstrated resolution of CAA at the first follow-up, even though they were not discharged with oral steroids.

Since the RAISE study^32^ showed the superiority of IVIG+MP over IVIG in preventing CAAs, diminishing the necessity for additional rescue treatment, and providing quick resolution of fever and inflammatory markers in severe KD, GC treatment with a wide dose range was implemented to prevent CAA and flare-up and to manage hyperinflammation in MIS-C.^21,28^ However, the RAISE study could not determine whether the reduction in CAA was caused by post-discharge 3 weeks steroid use or the combination therapy during the acute phase. Moreover, Son et al. in patients with MIS-C complicated by cardiovascular dysfunction, reported that shorter outpatient tapering (<3 weeks) of corticosteroids, had similar results to prolonged tapering time with the guide of CRP and ferritin levels.^27^ Additionally, it was found that longer GC use was associated with hyperglycemia and weight gain.^33^

TPE is an immunomodulatory treatment which provides rapid hemodynamic improvement and favorable changes in the cytokine profile in patients with hyperinflammatory status such as refractory KD,^34,35^ MAS,^36^ septic shock,^37^ MIS-C^38,39^ and COVID-19 disease.^40^ In a systematic review which included 953 patients with MIS-C, 3.8% needed ECMO.^41^ Only one patient (0.8%) in our cohort required ECMO, so early TPE, in addition to IVIG+MP + Anakinra, may prevent progression to ECMO by improving cardiovascular collapse in severest cases.

Thromboprophylaxis is recommended by the AHA for Kawasaki Disease,^8^ and the International Kawasaki Disease Registry also suggested prophylactic anticoagulation for MIS-C in patients at higher baseline risk for venous thromboembolism (e.g., patients ≥12 years old with altered mobility, obesity, known thrombophilia, history of thrombus, or critical presentation), alongside the use of pneumatic sequential compression devices.^42^ The immunopathogenesis of MIS-C involves the activation of platelet and coagulation cascades, which contribute to a hypercoagulable state.^43^ Thromboprophylaxis has shown to significantly improve thromboelastometry parameters in MIS-C.^44^ As part of our institutional approach, prophylactic enoxaparin has been used, and we have not encountered any major bleeding complications.

The retrospective nature of this analysis introduces several limitations. Firstly, the scaling of hyperinflammatory status may not comprehensively match the spectrum of cardiac dysfunction, and treatment selection criteria were occasionally adjusted based on the patient’s clinical conditions. Another limitation to this study was that varying durations between patients’ discharge and the first/second/third follow-ups introduce variability, rendering the longitudinal follow-up of laboratory values an estimation. Furthermore, some patients discontinued follow-ups, or their adherence was irregular. Lastly, it’s important to note that we couldn’t identify specific variants of SARS-CoV-2 viruses and investigate by distinguishing distinct waves during the study period.

As a conclusion, our study showed that titration of corticosteroids and anakinra day by day, with close follow-up of the hyperinflammatory status was really efficient in acute phase and long-term steroid use was unnecessary in the subacute phase of MIS-C. As one of the most important goals is the prevention of cardiac complications in MIS-C management, alongside rapid resolution of hyperinflammatory status, our findings suggest that short-term anakinra used during the acute phase is effective while the need for prolonged glucocorticoid therapy in the subacute phase is not clear.

## Supplementary information

Supplementary information

Supplementary table 2

Supplementary table 3

## Data Availability

The datasets generated and/or analyzed during the current study are not publicly available but are available from the corresponding author on reasonable request.
