# Supplementary material for: Multisystem Inflammatory Syndrome in Children with tailored therapy and six-month outcome
Source: Pediatr Res. 2026 Jan 10;100(2):765–75. doi: 10.1038/s41390-025-04706-6 (PMC13638463; doi:10.1038/s41390-025-04706-6)
Supplement: Supplementary file 1 [file 41390_2025_4706_MOESM1_ESM.docx]

**SUPPLEMENTAL FILE**

**Legends:**

**Supplemental Table S1.** Definitions of some clinical findings and organ involvement

**Supplemental Figure S1**. Case definitions of the Canadian Paediatric Surveillance Program (CPSP) and The Royal College of Paediatrics and Child Health (RCPHC) for Paediatric Inflammatory Multisystem Syndrome (PIMS), and the World Health Organization (WHO) and the Centers for Disease Control and Prevention (CDC) for Multisystem inflammatory Syndrome in Children (MIS-C), and coverage of the cohort with MIS-C on admission by these definitions on the Venn diagram

**Supplemental Figure S2**. The diagram of management in MIS-C.

**Supplemental Table S2.** The clinical and laboratory features of escalated therapies

**Supplemental Table S3.** The laboratory values of patients at throughout periods from admission to subsequent follow-ups

**Supplemental Table S4.** The features of patients with CAA

**Supplemental Figure S3.** The daily dose titration of anakinra during hospitalization.

**Supplemental Figure S4: A.** The daily dose titration of MP during hospitalization.

**B.** The titration of low-dose methylprednisolone and its effect on CRP levels during daily monitoring in patients treated with IVIG + low-dose MP.

**Supplemental Figure S5.** This classification and regression tree (CART) analysis showed treatment choices for MISC after the patients who underwent TPE were excluded.

**Supplemental Figure S6.** Day by day close monitoring of CRP, ferritin level and lymphocyte and thrombocyte count under dynamic MP and Anakinra doses in those who underwent TPE.

**Supplemental Figure S7.** The comparison of CRP, ferritin, ALC, thrombocyte at discharge and length of hospitalization of treatment modalities which was selected for subanalysis.

**Supplemental Figure S8.** The comparison of CRP, ferritin, thrombocyte, ALC and length of hospitalization in those who underwent TPE with low dose steroid and high dose steroid and altogether.

**Supplemental Table S1.** Definitions of some clinical findings and organ involvement

| **Clinics and Organ Involvement** | **Definition** |
| --- | --- |
| Cardiac abnormality | Arrhythmia, shock, hypotension or LV ejection fraction of <55%, valve insufficiency, pericardial effusion, coronary artery abnormality, increased troponin and/or BNP |
| Decreased left ventricular (LV) function | LV ejection fraction of <55%. |
| Coronary artery abnormality | Boston z score system: normal <2, dilatation >2 to <2.5, and aneurysm >2.5 (1) |
| Gastrointestinal involvement | Abdominal pain, diarrhea, vomiting, pericholecystic edema, mesenteric lymphadenopathy, intrabdominal free fluid, increased thickness of terminal ileum and diameter of appendix vermiformis, hepatitis |
| Renal involvement | Acute kidney injury, proteinuria, or hematuria. |
| Acute kidney injury | Increased by 1.5-1.9 times baseline within 7 days OR increase by >0.3 mg/dL within 48 hour OR decreased urine output less than 0.5 mL/kg/h for 6-12 hours (2) |
| Neurologic involvement | Seizure, headache, altered mental status, encephalitis, meningism/aseptic meningitis |
| Macrophage activation syndrome (MAS) | 2016 Classification Criteria for Macrophage Activation Syndrome Complicating Systemic Juvenile Idiopathic Arthritis was used for diagnosis of MAS: In a febrile patient with MIS-C (instead of originally systemic juvenile idiopathic arthritis); Ferritin > 684 ng/ml and any of 2 following criteria: platelet > 181 x 10^9/L, AST > 48 U/L, Triglycerides > 156 mg/dl, Fibrinogen < 360 mg/ml |
| Bicytopenia | ALC< 1000 /mm^3^ and Platelet < 150 /mm^3^ |

**Supplemental Figure S1**. Case definitions of the Canadian Paediatric Surveillance Program (CPSP) and The Royal College of Paediatrics and Child Health (RCPHC) for Paediatric Inflammatory Multisystem Syndrome (PIMS), and the World Health Organization (WHO) and the Centers for Disease Control and Prevention (CDC) for Multisystem inflammatory Syndrome in Children (MIS-C), and coverage of the cohort with MIS-C on admission by these definitions on the Venn diagram

**Supplemental Figure S2. The diagram of management in MIS-C.** Based on the clinical severity of patients with Multisystem Inflammatory Syndrome in Children (MIS-C), as assessed by hyperinflammatory status with high CRP and Ferritin level and low platelet and lymphocyte count, presence of macrophage activation syndrome, number of organ involvements, the spectrum of cardiac dysfunction with myocarditis, and the need of cardio/pulmonary support, treatment initiation followed immediately the diagnosis. The initial treatments for timely administration were Intravenous Immunoglobulin (IVIG), IVIG + low-dose Methylprednisolone (MP), or IVIG + low-dose MP + Anakinra.

Escalation performed for patients not responding to the initial treatment within 24 hours, especially after 12 hours of IVIG administration. Clinical response with laboratory investigation guided decisions for escalation as well. In the absence of clinical improvement, involving parameters like fever, hypotension, and laboratory values, low-dose MP was escalated to pulse, and/or Anakinra was added. Daily echocardiographic monitoring in patients with shock and monitoring in cardiac markers as well as hyperinflammatory status played a crucial role in deciding the necessity of Therapeutic Plasma Exchange (TPE).

**Supplemental Table S4.** The features of patients with CAA

| **Patients with CAA** | **Age (Gender)** | **Phenotype** | **ECHO at hospitalization** | **ECHO at 1^st^ follow-up** | **ECHO at 2^nd^ follow-up** | **ECHO at 3^rd^ follow-up** | **Immuno-modulatory treatment** | **Post discharge steroid** | **ASA** | **Enox-aparin** |
| --- | --- | --- | --- | --- | --- | --- | --- | --- | --- | --- |
| P1 | 3 (M) | Shock | Aneurysm | Normal |  |  | IVIG + MP + Anakinra + TPE | No | Yes | Yes |
| P2 | 6 (F) | F&I | Aneurysm | Normal |  |  | IVIG + MP + Anakinra | No | Yes | Yes |
| P3 | 9 (M) | KD | Dilatation | Normal | Normal | Normal | IVIG + MP + Anakinra | No | Yes | Yes |
| P4 | 14 (M) | KD | Dilatation | Normal | Normal | Normal | IVIG + MP + Anakinra | No | Yes | Yes |
| P5 | 10 (M) | KD | Dilatation | Normal | Normal |  | IVIG + (HD) MP + Anakinra | No | Yes | Yes |
| P6 | 13 (M) | KD | Dilatation | Normal |  |  | IVIG + (HD) MP + Anakinra | No | Yes | Yes |
| P7 | 4 (F) | KD | Dilatation | unknown |  |  | IVIG + MP | No | Yes | Yes |
| P8 | 1 (M) | KD | Normal | Dilatation | unknown |  | IVIG + MP | No | Yes | Yes |
| P9 | 6 (M) | KD | Normal | Dilatation | Normal | Normal | IVIG + MP | No | Yes | Yes |

**Supplemental Figure S3.** The daily dose titration of anakinra during hospitalization.

**Supplemental Figure S4. A. The daily dose titration of MP during hospitalization.**

**B. The titration of low-dose methylprednisolone and its effect on CRP levels during daily monitoring in patients treated with IVIG + low-dose MP.** CRP monitorization was used to taper IVMP throughout hospitalization. The same titration was conducted for Anakinra; however, analysis was not performed on the other clusters because the normalization of CRP cannot be specifically attributed to either the Anakinra titration or the IVMP titration.

**Supplemental Figure S5. This classification and regression tree (CART) analysis showed treatment choices for MISC after the patients who underwent TPE were excluded.** TPE was the highest level of escalation among immunomodulatory treatments, so the patients who received TPE were subtracted from the decision tree for high dose versus low dose MP. A platelet count was added to the y axis to depict the high dose or low dose to terminal nodes. On the right branch, patients with shock were stabilized almost half as much with IVIG + low dose MP + Anakinra as with IVIG + high dose MP + Anakinra. If the patient did not have shock and had an INR over 1.32, IVIG + high dose MP + Anakinra was given. IVIG + low dose MP were administered to patients without shock whose INR was less than 1.32 and lymphocyte count was less than 1.75; however, additional thrombocytopenia (and therefore bicytopenia) required Anakinra. If neither thrombocytopenia nor lymphopenia were present (Node 4 and Node 5), the treatment was generally not escalated to high dose MP or Anakinra. (Abbreviatons: Y; Yes, N;No) [Red point= MP + Anakinra, Blue Point= Only MP)

**Supplemental Figure S6.** **Day by day close monitoring of CRP, ferritin level and lymphocyte and thrombocyte count under dynamic MP and Anakinra doses in those who underwent TPE.**

Day 1 stands for just before TPE and Day 2 stands for next day of TPE, so the TPE session which was plotted in this figure for all patients was performed between Day1 and Day2. For this figure, patients who were performed the TPE on in multiple sessions and the patient who underwent ECMO were excluded. The decrease in CRP and ferritin following TPE in Day 2 was greater than the other treatments, however there was a slight elevation in CRP and Ferritin between Day 3 and Day 4 as a rebound inflammation.

**Supplemental Figure S7.** The comparison of CRP, ferritin, ALC, thrombocyte at Day 4 and length of hospitalization of treatment modalities which was selected for subanalysis.

**Supplemental Figure S8.** The comparison of CRP, ferritin, thrombocyte, ALC at Day 4 and length of hospitalization in those who received TPE with low dose steroid and high dose steroid.

**eReference**

1. McCrindle BW, Rowley AH, Newburger JW, Burns JC, Bolger AF, Gewitz M, et al. Diagnosis, Treatment, and Long-Term Management of Kawasaki Disease: A Scientific Statement for Health Professionals From the American Heart Association. Circulation. 2017;135(17):e927-e99.
2. Section 2: AKI Definition. Kidney International Supplements. 2012;2(1):19-36.
